# The visual gamma response to faces reflects the presence of sensory evidence and not awareness of the stimulus

**DOI:** 10.1098/rsos.150593

**Published:** 2016-03-02

**Authors:** Gavin Perry

**Affiliations:** Cardiff University Brain Imaging Centre (CUBRIC), School of Psychology, Cardiff University, 70 Park Place, Cardiff CF10 3AT, UK

**Keywords:** magnetoencephalography, gamma-band, oscillations, awareness, face perception

## Abstract

It has been suggested that gamma (30–100 Hz) oscillations mediate awareness of visual stimuli, but tests of this hypothesis have produced differing results. We used phase scrambling to vary the perceptibility of face stimuli in order to determine whether gamma is indeed linked to perceptual awareness. Magnetoencephalography was used to measure the gamma response in 25 participants while viewing three conditions in which faces were presented either above, below or at the threshold for detection. In each of 400 trials (100 each for the sub- and suprathreshold conditions, 200 for the threshold condition), participants indicated whether they perceived a face in the stimulus. Gamma-band activity during the task was localized to bilateral ventral occipito-temporal cortex. For the threshold condition, we failed to find a significant difference in gamma amplitude between trials in which a face was perceived relative to those in which no face was perceived. However, we did find that gamma amplitude was significantly increased for threshold relative to subthreshold stimuli and for suprathreshold relative to threshold stimuli. This leads us to conclude that the gamma response to faces is primarily modulated by the presence of sensory evidence of a face rather by perceptual awareness.

## 1. Background

Neuronal oscillations at gamma (greater than 30 Hz) frequencies appear to be a common feature of cortical electrophysiology during processes of perception, cognition and action (see [1,2] for two recent reviews). However, despite over two decades of investigation of the phenomenon, the functional role of this oscillatory response is still unclear (see [3–5] for suggestions about the possible functional significance of gamma oscillations).

One early proposal was that gamma oscillations mediate perceptual awareness [6], and this still remains a focus of research. Evidence in favour of this hypothesis comes from studies in which various techniques such as backward masking [7–9], presentation of stimuli at the detection threshold [10], the attentional blink [11] and dichoptic fusion [12] have demonstrated a link between perceptual awareness and the amplitude of visual gamma oscillations. However, not all evidence agrees with this conclusion. In a study in which visual awareness was altered by the combination of image noise and stimulus pre-exposure, the gamma response did not reflect perceptual awareness, but instead was sensitive to the amount of sensory evidence present in the image [13]. Conversely, a recent study which used inattentional blindess to modulate visual awareness found evidence that gamma amplitude may in fact be linked to the task-relevance of target stimuli [14]. Thus, it remains unclear whether there is a general link between the visual gamma response and perceptual awareness.

In order to disentangle the contributions of perceptual awareness and sensory evidence to the gamma response, we designed an experiment in which both aspects of a stimulus could be probed using a single parameter. To achieve this, we varied the perceptibility of face stimuli by using the ‘phase scrambling’ method in which the spatial phase of the stimulus is systematically varied by the addition of noise [15–17]. This method of varying stimulus perceptibility was chosen as it ensures that the Fourier amplitude spectra of test stimuli are matched across conditions, and eliminates the possibility that any differences in the gamma response between conditions could be due to low-level image properties (i.e. the first- and second-order image statistics).

Magnetoencephalography (MEG) was used to measure the gamma response while individuals viewed three stimulus conditions in which faces presented above, below or at the detection threshold. By contrasting threshold trials in which the face was perceived with those in it which it was not perceived, we could test the effects of perceptual awareness for a fixed level of phase noise. By contrasting trials in which the face was perceived between the threshold and suprathreshold condition, and by contrasting trials in which the face was not perceived between the threshold and subthreshold conditions, we could test the sensitivity of the gamma response to the level of phase noise both when the face did or did not enter perceptual awareness.

## 2. Material and methods

### 2.1 Participants

Participants were 25 volunteers (eight males; age range: 18–52 years, mean: 25 years) with normal or corrected-to-normal vision (based on self-report).

### 2.2 Stimuli

Face stimuli were 40 images each showing a frontal view of one of 20 females and 20 males, taken from the ECVP Utrecht face set (http://pics.psych.stir.ac.uk/zips/utrecht.zip). Images were cropped to 450×600 pixels, centred on a point midway between the eyes. Images were converted to greyscale and normalized by averaging the two-dimensional Fourier amplitude spectra. Images were further scaled so that mean pixel intensity was equal to the display background and the standard deviation of pixel intensity was equal to 20% of the displayable range. For each participant, the stimuli were randomly assigned into four groups of 10, so that a different set of stimuli were used in each session/condition for each run of the experiment (see below). A further four images from the same stimulus set were similarly processed for use in practice trials.

During the experimental procedure, varying levels of phase scrambling were applied to the images according to the ‘weighted mean phase’ method [16] in which noise is added to the spatial phase of each image according to a weighting factor, *w*, in the range [0,1]. This weighting factor determines the proportion of the original image phase that is retained in the scrambled image.

### 2.3 Experimental procedure

**Figure 1.**
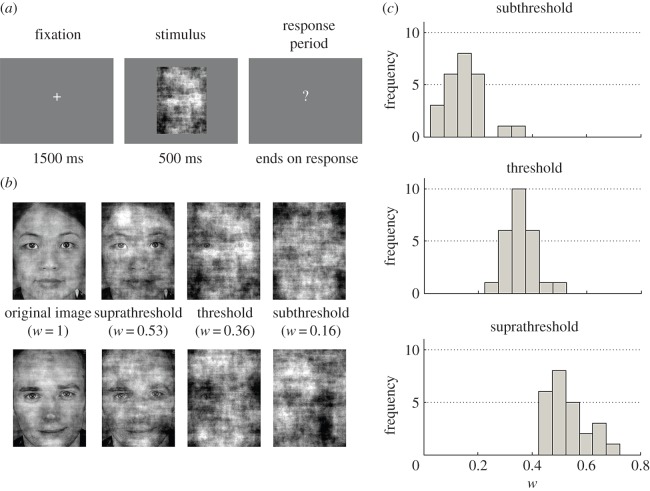
(*a*) Illustration of the timecourse of an individual trial. Each trial was separated by a blank screen of 1000 ms duration. (*b*) Examples of two stimuli at the levels of phase noise used for each condition (values of *w* used are based on the average of the values used in each condition across participants). Original unscrambled images (shown after averaging of Fourier amplitudes across stimuli) are also shown. (*c*) Histograms showing the distribution across participants of the values of *w* (the proportion of the phase from the original image retained in the scrambled image) estimated from psychometric fits to produce a face detection rate of 0.5% (subthreshold), 50% (threshold) and 99.5% (suprathreshold).

The experimental procedure consisted of presenting participants with face stimuli at different levels of phase scrambling. Each trial followed a set procedure in which a central fixation cross was presented for 1500 ms followed by the stimulus for 500 ms (figure 1*a*). At stimulus offset, a question mark appeared and remained on-screen until the participant made a response. Following a response, the screen was blank for 1000 ms and the next trial then began.

Participants were informed that a face may or may not be present in each stimulus that they saw, and that when the question mark appeared on screen they had to respond as to whether or not a face had been present in the immediately preceding stimulus. Responses were made by pressing one of two buttons with their right hand.

All stimuli were displayed centrally on a median-level grey background (luminance: 26.5 cd m^−2^) using a gamma-corrected Mitsubishi Diamond Pro 2070 CRT monitor with a screen resolution of 1024×768 pixels and a refresh rate of 100 Hz. The monitor was positioned outside the magnetically shielded room and viewed through an aperture in the wall. The viewing distance was 2.1 m, with stimulus images subtending 4.69°×6.25° of visual angle. The experimental paradigm was implemented in Matlab (The Mathworks, Inc., Natick, MA, USA) using the Psychophysics Toolbox [18–20].

### 2.4 Experimental design

The overall design of the study was to present participants with stimuli at levels of phase scrambling corresponding to the threshold for detection, or at fixed intervals above and below this threshold. This required participants to take part in three consecutive sessions of testing.

Participants initially underwent a practice session in which they viewed 12 trials consisting of each of the four practice stimuli at three levels of phase noise (*w*=0, 0.3, 0.6). This was followed by a session in which each participant’s psychometric function for detecting the face stimuli under varying levels of phase noise was determined using a method of constant stimuli. During this session, participants underwent 140 trials in which each of the first set of 10 randomly selected face stimuli were presented twice at seven levels of scrambling: *w*=0, 0.2, 0.25, 0.3, 0.35, 0.4, 0.45, 0.5, 0.7. Detection rates at each level of scrambling were then fit for each participant with a Weibull function. This fit was used to estimate the values of *w* required to produce face detection rates of 0.5, 50 and 99.5%.

Finally, in the MEG session, these three values of *w* were used to set the level of phase noise in three conditions, respectively referred to as ‘subthreshold’, ‘threshold’ and ‘suprathreshold’ from here on (see figure 1*b* for examples of these conditions). Participants underwent 100 trials each of the subthreshold and suprathreshold conditions, and 200 trials of the threshold condition. The three remaining sets of 10 face stimuli not used in the previous session were each assigned to one of the three conditions, with each stimulus being presented in only a single condition per participant (this was done to prevent increased familiarity with faces presented in the suprathreshold condition from enhancing detection rates in the other two conditions as the experiment progressed). Trials in which the participant took longer than 1 s to respond after stimulus offset were excluded from analysis.

During this session, whole-head MEG recordings were made using a 275-channel CTF radial gradiometer system sampled at 600 Hz. Data were recorded in 2.5 s epochs beginning at 1 s prior to stimulus onset. An additional 29 reference channels were recorded for noise cancellation purposes, and the primary sensors were analysed as synthetic third-order gradiometers [21].

### 2.5 Data analysis

During the MEG recording session, participants’ head position was continuously localized by means of three fiducial coils attached to specific anatomical locations on the scalp (nasion and left and right pre-auricular). The mean position of each coil was calculated across all data samples, and trials in which any coil was displaced from its mean position by greater than 1 cm were excluded from analysis. Data were additionally inspected visually and trials containing excessive muscle artefacts were also excluded.

Each participant had a structural magnetic resonance imaging (MRI) scan at 1 mm isotropic voxel resolution for use with MEG source analyses. The anatomical locations of the fiducial coils were verified for each participant using high-resolution digital photographs and were then located on each participant’s MRI. A multiple local spheres forward model [22] was derived by fitting spheres to the individual’s brain surface extracted from their MRI using FSL’s Brain Extraction Tool [23].

For analysis of the gamma response, data were bandpass filtered using a fourth-order bi-directional Butterworth filter at 50–90 Hz (this choice of bandpass was based on the findings of Perry & Singh [24]). Source analysis was performed using synthetic aperture magnetometry (SAM) [25]. Data covariance matrices were calculated from the concatenation of all trials regardless of condition (but excluding trials containing artefacts). In order to localize face-specific responses, data from the ‘subthreshold unseen’ and ‘suprathreshold seen’ conditions were projected into each participant’s source-space at 4 mm isotropic resolution. For each of the two conditions, the Hilbert transform was used to produce the analytic signal of the filtered data, and the mean amplitude of the response was calculated during the time period of 100–500 ms, after baseline correction against the −500 to −100 ms time period. Statistical parametric maps of the contrast between these two conditions were then generated for each participant based on Welch’s *t*-statistic.

Locations of interest for virtual sensor analyses were identified for each analysis by finding image peaks in ventral occipito-temporal regions of individual participants’ *t*-statistical images. Where present, one peak was found for each cortical hemisphere. Sensor-level data were then spatially filtered using the beamformer at the locations of interest to create a single timeseries per trial per peak per participant. Spectrograms were calculated for each timeseries by bandpassing the data using filters of 4 Hz bandwidth centred on 40–100 Hz in 1 Hz steps and using the Hilbert transform to compute the amplitude of the analytic signal at each frequency step. Time–frequency data were then averaged across all trials of a given condition for each participant, and the magnitude of the response was calculated as the percentage amplitude change relative to the mean amplitude during the baseline period (−500 to −100 ms). A single summary statistic of the gamma response in each condition was calculated for each participant by averaging across the 100–500 ms time window and the 50–90 Hz frequency range.

We additionally performed volumetric whole-brain analyses of each of our pairwise contrasts of interest (‘threshold seen’ versus ‘threshold unseen’, ‘suprathreshold seen’ versus ‘threshold seen’, and ‘subthreshold unseen’ versus ‘threshold unseen’) in order to test for the possibility that the virtual sensor analysis missed significant differences between conditions in other brain regions or at other frequencies.

For these analyses, we generated SAM images (based on response magnitude within the time and frequency windows given above) of *t*-statistical contrasts between specific pairs of conditions for each participant. These were normalized to a template brain using FSL’s Flirt software, then used to create second-level *t*-statistical images of the difference of each voxel from zero (i.e. against a null hypothesis of no difference between conditions). In addition to testing at the 50–90 Hz frequency band used in the paper, we also repeated the tests in the following sub-bands of the gamma frequency range: 30–50, 40–60, 50–70, 60–80, 70–90 and 80–100 Hz.

For each of these whole-brain contrasts, voxelwise *p*-values were calculated using permutation testing (based on 5000 permutations per test) and were corrected for multiple comparisons at the whole-brain level by testing against the omnibus null hypothesis using the maximum test statistic [26].

## 3. Results

### 3.1 Psychometric data

**Figure 2.**
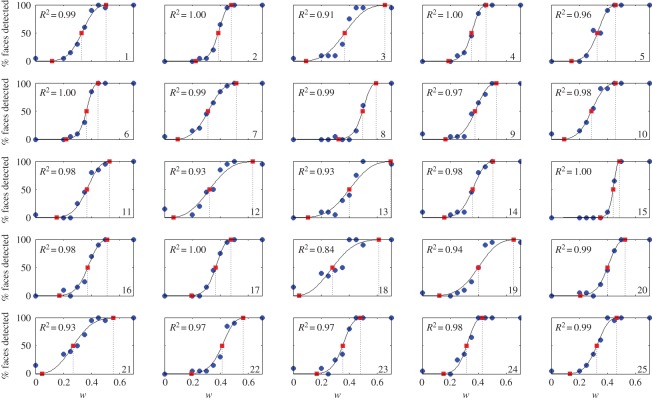
Plots of psychometric functions of face detection for varying levels of scrambling in each of 20 participants. *w*—the proportion of the original image phase included in the scrambled image—is given on the *x*-axis, the percentage of faces detected is given on the *y*-axis. Blue points show the actual data for each participant, the black line shows the best fitting Weibull function (*R*^2^ for this fit is given in each plot), and the red points and dotted lines show the values of *w* used for the subthreshold, threshold and suprathreshold conditions.

For each participant, we used a method of constant stimuli to determine the psychometric function for face detection under varying levels of phase noise. Each participant’s data were fit with a Weibull function (*R*^2^≥0.84 for all fits; figure 2) which was then used to determine the level of phase scrambling, *w*, required to produce face detection rates of 0.5, 50 and 99.5% for each participant (figure 1*c*).

### 3.2 Magnetoencephalography data

**Figure 3.**
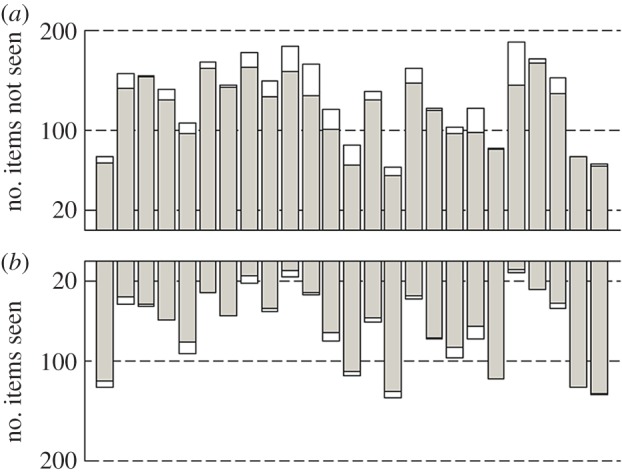
Bar charts showing the number of trials in which each participant reported seeing (*b*) or not seeing (*a*) a face in the threshold condition. Shaded areas show the number of trials remaining after trial exclusions.

During MEG recording, participants were presented with face images which were phase scrambled at the three levels of *w* determined from their psychometric functions. These are defined ‘subthreshold’, ‘threshold’ and ‘suprathreshold’ conditions, respectively. For analysis, trials were further divided into subconditions of ‘seen’ and ‘unseen’ depending on whether the participant had perceived the presence of a face in that trial or not. The small number of trials in the ‘subthreshold seen’ and ‘suprathreshold unseen’ conditions were discarded, leaving four conditions for analysis: ‘subthreshold unseen’, ‘threshold unseen’, ‘threshold seen’ and ‘suprathreshold seen’. Although the level of phase scrambling in the threshold condition had been determined such that participants should, on average, perceive the presence of a face on 50% of occasions (and thus lead to equal number of trials on average in the ‘threshold unseen’ and ‘threshold seen’ conditions) we in fact found a bias across the group for faces to be more likely to be unseen in the threshold condition (figure 3). This meant that in the case of three individuals there were 20 or fewer trials in the ‘threshold seen’ condition after exclusions, and for that reason those participants were excluded from further analysis.

**Figure 4.**
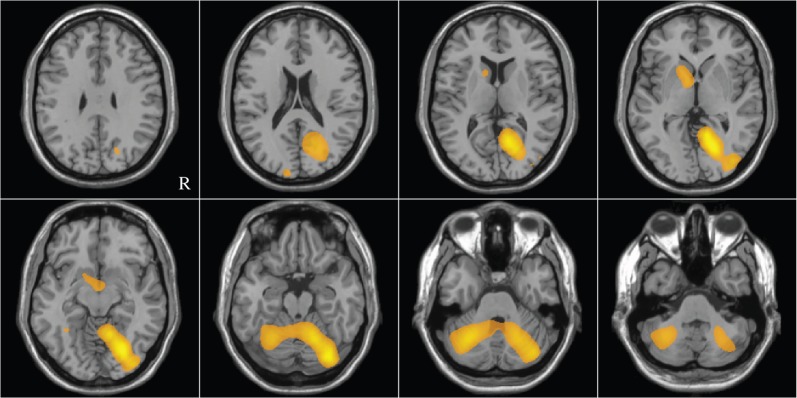
Cortical slices taken from the template brain. The overlay shows the group mean of *t*-statistical contrasts between the ‘suprathreshold seen’ and ‘subthreshold unseen’ conditions (overlay is arbitrarily thresholded at 0.6 for illustrative purposes only).

Face-specific gamma responses were source-localized for each individual using the contrast between the ‘subthreshold unseen’ and ‘suprathreshold seen’ conditions (see figure 4 for the group average image). This contrast was used as it incorporates differences both in levels of phase noise (subthreshold versus suprathreshold) and in awareness (unseen versus seen) and hence any sources of interest found would have been unbiased with respect to whether they were due to either (or both) of these differences. Image peaks were identified in ventral occipito-temporal cortex in both hemispheres, where present (an identifiable peak was found for 20 participants in the left hemisphere and 17 participants in the right hemisphere: group mean Talairach coordinates were −35.4, −74.3, −10.0 mm and 40.3, −69.67, −7.5 mm, respectively).

**Figure 5.**
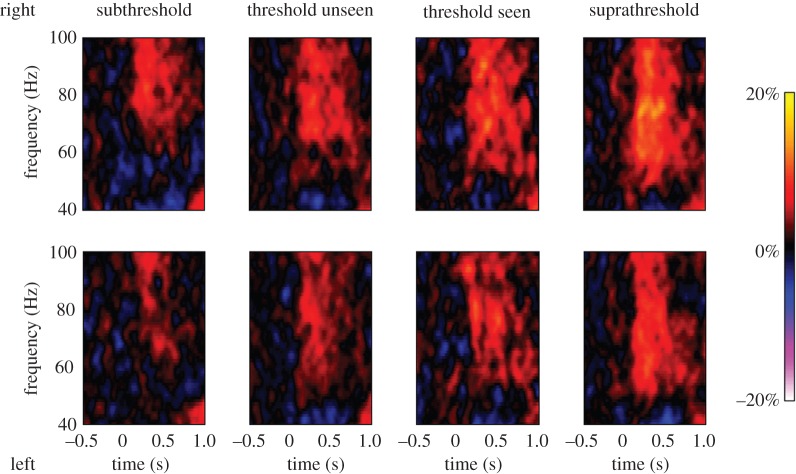
Group average spectrograms of virtual sensor responses from each of four conditions for each hemisphere (the plots labelled subthreshold and suprathreshold show the ‘subthreshold unseen’ and ‘suprathreshold seen’ conditions). The colour scale represents percentage change from baseline.

**Figure 6.**
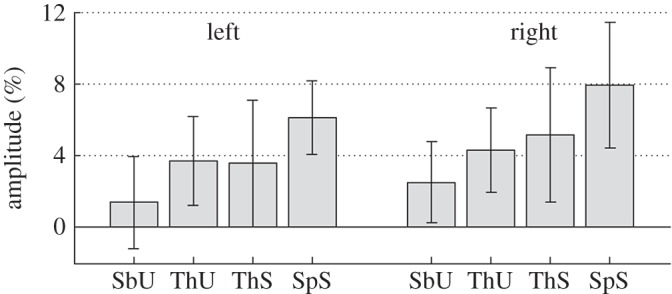
Group mean (±s.d.) gamma amplitude (% change relative to baseline) for each of the four conditions in each hemisphere. SbU, subthreshold unseen; ThU, threshold unseen; ThS, threshold seen; SpS, suprathreshold seen.

Spectrograms of the response at these locations were generated for each condition (figure 5). The magnitude of the gamma response in each condition was characterized as the average response across the 100–500 ms time window and the 50–90 Hz frequency range (figure 6). We performed a series of planned pairwise comparisons between conditions using paired, two-tailed *t*-tests (as tests were repeated for both hemispheres, the significance level used for each test was Bonferroni corrected from *α*=0.05 to 0.025 and confidence intervals were calculated at the 97.5% level; effects sizes are given by Cohen’s *d*).

Firstly, to test if the gamma response was sensitive to whether or not the face was perceived for a fixed level of phase noise, we compared the ‘threshold seen’ condition with the ‘threshold unseen’ condition. We did not find any significant differences between these conditions (right hemisphere: *t*_16_=1.3, *p*=0.35, CI [−1.4,3.1], *d*=0.24; left hemisphere: *t*_19_=−0.3, *p*=0.8, CI [−1.6,1.3], *d*=−0.05).

Secondly, to test if the gamma response was sensitive to the amount of phase noise in trials in which the face was perceived, we compared the ‘suprathreshold seen’ and ‘threshold seen’ conditions. The response to suprathreshold stimuli was significantly greater than that to the threshold stimuli in both hemispheres (rh: *t*_16_=3.1, *p*=0.007, CI [0.6,4.9], *d*=0.75; lh: *t*_19_=4.0, *p*=7×10^−4^, CI [1.0,4.1], *d*=0.90).

Finally, we were interested to see whether the gamma response was sensitive to the level of phase noise even when no face was perceived in the image. Thus, we contrasted the ‘threshold unseen’ with ‘subthreshold unseen’ conditions. In both hemispheres, the gamma response was greater to the threshold than to the subthreshold stimuli (rh: *t*_16_=4.1, *p*=9×10^−4^, CI [0.7,2.8], *d*=0.99; lh: *t*_19_=4.9, *p*=1×10^−4^, CI [1.2,3.5], *d*=1.10).

We additionally performed volumetric whole-brain analyses of each of our pairwise contrasts of interest (‘threshold seen’ versus ‘threshold unseen’, ‘suprathreshold seen’ versus ‘threshold seen’ and ‘subthreshold unseen’ versus ‘threshold unseen’) at a variety of different sub-bands of the gamma frequency range (see Material and methods), in order to test for the possibility that the virtual sensor analysis missed significant differences between conditions in other brain regions or at other frequencies. However, we did not find significant differences at *α*=0.05 for any of the three contrasts at any frequency band.

## 4. Discussion

In this study, we set out to test the extent to which the gamma response to faces reflects both sensory evidence and perceptual awareness. By varying the degree of phase scrambling applied to face stimuli around the detection threshold and measuring the resulting gamma responses, we have produced two main findings. Firstly, when stimuli were presented at the threshold for detection we did not find any evidence that the gamma response was dependent on whether a face was perceived in the stimulus or not. Secondly, we found that the gamma response increases with decreasing phase noise both in circumstances where the face is perceived, but notably also in circumstances where participants did not report perceiving a face. Therefore, the evidence of this study strongly suggests that the amplitude of the gamma response is coupled to the amount of evidence for the presence of a face in the stimulus (as modulated by the level of phase noise in this instance).

We did not find any evidence supporting the hypothesis that the gamma response is related to awareness of the face. While an absence of evidence does not constitute evidence of absence *per se*, the fact that we were able to show significant differences in gamma response with changes in phase noise demonstrates that our experimental design could reveal differences in gamma amplitudes where such differences exist. This makes it less likely that any lack of effect due to differences in awareness could be attributed to poor signal-to-noise ratio of the gamma signal. Post-hoc power calculations suggest that the study had statistical power of 0.8 to show differences in the ‘threshold seen’ versus ‘threshold unseen’ contrast of 1.99% and 2.97% in the left and right hemisphere, respectively (these correspond to effect sizes of *d*=0.74 and 0.81). Conversely, the largest difference between conditions we found in any of our three planned comparisons were 2.56% and 2.75% in the left and right hemispheres, respectively (in both cases in the ‘threshold seen’ versus ‘suprathreshold seen’ contrast). Post-hoc power calculations suggest the study had power of 0.96 and 0.73 (in the left and right hemispheres, respectively) to find differences of this size in the ‘threshold seen’ versus ‘threshold unseen’ contrast. Thus, we had sufficient power to find large effects of perceptual awareness but may have lacked power to find more subtle differences between the ‘threshold seen’ and ‘threshold unseen’ conditions (particularly in the right hemisphere). This suggests that while we cannot conclude that differences in gamma amplitude due to awareness do not exist, if they do exist they are likely to be small relative to the differences found due to changes in sensory evidence.

It should be noted, however, that the scope of the current study is limited to the face-specific gamma response found previously by Perry & Singh [24]. This was due to the fact that times, frequencies and locations of interest were defined by those found in that previous study. This was purposeful, as it meant that our analysis was unbiased with respect to whether the effect of interest could be due to sensory evidence or awareness; in the original Perry & Singh study, in which unscrambled faces were compared to fully scrambled faces (i.e. with *w*=0), both sensory evidence and awareness of a face differed between conditions, and so either or both of these variables could have accounted for the effect. Likewise, the contrast between ‘suprathreshold seen’ and ‘subthreshold unseen’ used as a localizer in this study could have been driven by effects of sensory evidence (suprathreshold versus subthreshold) or awareness (seen versus unseen) or both, and therefore was also unbiased with respect to the source of the contrast. The limitation of this approach, however, is that we cannot preclude the possibility of their being other gamma responses present during the experiment at other frequencies and/or locations which were linked to awareness. We did perform whole-brain non-parametric tests of each of our paired effects of interest (‘threshold seen’ versus ‘threshold unseen’, ‘suprathreshold seen’ versus ‘threshold seen’ and ‘subthreshold unseen’ versus ‘threshold unseen’)—both at 50–90 Hz and at other sub-bands of the gamma frequency range—in order to locate any possible effects of awareness (or additional effects of sensory evidence that we may have missed), but we did not find any significant differences. While these null effects cannot provide evidence for an absence of any effect, they did preclude us from identifying any further locations and/or frequencies of interest for further analysis.

One further limitation of the study design is that, as we did not include separate behavioural probes of psychophysical performance and awareness, we cannot dissociate between participants’ behavioural thresholds for responding and the underlying perceptual thresholds for awareness. It should be noted that some participants appeared to use different thresholds for detection between the initial testing session and the MEG recording session. In the latter session, many participants showed a bias in the threshold condition to reporting the face as unseen despite phase noise in that condition being set at a level that should have led participants to see the face approximately 50% of the time based on responses in the first session. This suggests that at least some of the participants varied their behavioural thresholds for face detection between sessions, indicating that these thresholds were not fixed but varied depending on context. It is not clear if this reflects changes in the criteria for awareness or changes in the criteria for responding, and to the extent that those criteria may vary separately, the results of this study only pertain to the latter. Only more sophisticated study designs in which response thresholds and thresholds to awareness can be probed separately can elucidate these potential difficulties.

The findings of this study stand in contradiction to a number of previous studies which have found evidence that gamma is linked to perceptual awareness [7–12], but is in agreement with at least two previous studies that have failed to demonstrate this link [13,14]. It is notable that these previous studies have used a variety of different methodologies, and thus it seems reasonable to conclude that the apparent link between gamma oscillations and awareness (or the lack thereof) may be dependent on the specific method used. One common factor in many, but not all, of the studies reporting a link between gamma and awareness is that in those studies it was the awareness of the presence of a target stimulus that was tested (e.g. [7–11]; but notably not the dichoptic fusion task of Fahrenfort *et al.* [12]). In this study (and in the two previous studies that failed to find a relationship between gamma and awareness [13,14]), participants were always aware of the presence of a stimulus on each trial, and it was instead awareness of some meaningful structure within the stimulus that was modulated (and indeed a similar interpretation could also be applied to the results found by Fahrenfort *et al.* [12]). Thus, if gamma is sensitive to both the awareness of the presence of a stimulus and to the perceptual content of that stimulus, then it would be possible to reconcile the apparent conflict in the results of this study with those other earlier studies.

One further complicating factor in comparing this with other studies is that there is some diversity across studies in the time and frequency windows in which gamma is measured. This is critically important as it has been established that some components of the visual gamma response may reflect (or at least be coupled to) local spiking activity rather than gamma oscillations [27–29], and thus studies using different definitions of gamma may be measuring different aspects of the underlying neurophysiological response. The peaked and relatively narrow-band nature of the signal measured here (as demonstrated by Perry & Singh [24]) would suggest that this response is oscillatory in nature, but we cannot definitely state that this was the case. An additional factor to be considered is that signals in gamma frequency measured in electroencephalography (EEG) can sometimes reflect artefacts due to the effects of eye movements on the reference channel [30], and this may have impacted on previous studies (many of which used EEG). As MEG is reference free, and as we performed our analysis after source localization to ventral occipto-temporal cortex, it is unlikely that our data were affected by eye-movement-related artefacts. However, as we did not measure eye movements (either in this study or in Perry & Singh [24]) we cannot definitively state that they do not play a modulatory role in the gamma-band response measured here, and this question should be addressed in a future study.

The application of smoothed white noise (applied in the image domain) to stimuli by Aru *et al.* [13] is the closest analogue to the current task in any previous study. They found that gamma amplitude was sensitive to image noise but not to the level of pre-exposure of the stimuli, despite both parameters modulating awareness, leading to the conclusion that gamma is sensitive to the sensory evidence present in the stimulus but not to perceptual awareness. One potential limitation of that previous study is that the noise process used leads to changes in the spatial frequency content of the stimuli and it may have been this factor—rather than changes in the sensory evidence—that explains their results. By using a noise process (phase scrambling) which preserves the amplitude spectra of the stimuli, we have excluded this possibility, and strengthened the evidence in favour of the gamma response being sensitive to sensory evidence.

The results of the current study, therefore, are in agreement with this previous study while also demonstrating a complementary effect: where Aru *et al.* [13] found that awareness can be modulated without altering the gamma response, we have demonstrated that it is possible to produce changes in gamma without corresponding changes in awareness (and in fact even in the absence of any awareness of the target stimulus whatsoever). Taken together the two experiments show that the gamma response can be fully dissociated from perceptual awareness.

Indeed the data in the current study could be explained by a simple model in which the gamma generating region(s) integrates input from a population of cells each acting as a detector for one or more facial features, and for which the amplitude of the gamma simply reflects the strength of this input activity. Such a model would be consistent with the proposal of Merker [5] that the strength of gamma within a cortical area merely reflects the level of ‘balanced tension’ between local excitatory and inhibitory processes under afferent drive, and that gamma oscillations themselves may not have any high-level functional role (such as underpinning perceptual awareness).

Consequently, the fact that gamma amplitude did not appear to differ in the current study depending on whether or not a face was actually seen—but, based on previous evidence outlined above, that gamma appears to be sensitive to whether or not *any* stimulus reaches awareness—suggests that gamma may be a necessary but not sufficient condition for perceptual awareness to occur. However, we suggest that this conclusion may merely reflect the fact that activation of the cortical region involved in processing a specific class of stimulus is necessary (but not in itself sufficient) for that stimulus to enter perceptual awareness.

## Data accessibility

The data supporting this article can be accessed at http://dx.doi.org/10.6084/m9.figshare.1588737.
